# Seasonal variations in suicide mortality in Canada: A nationwide analysis

**DOI:** 10.17269/s41997-026-01155-4

**Published:** 2026-02-04

**Authors:** Emily Lau, Rebecca Plouffe, Li Liu, Gisèle Contreras, Karin Johnson, Geneviève Gariépy

**Affiliations:** 1 Public Health Agency of Canada, Ottawa, ON Canada; 2 Dalla Lana School of Public Health, University of Toronto, Toronto, ON Canada

**Keywords:** Seasonal variations, Suicide, Population health, Canada, Variations saisonnières, Suicide, Santé de la population, Canada

## Abstract

**Objective:**

To investigate the seasonality of suicide mortality in Canada and explore differences across sex and age.

**Methods:**

We analyzed monthly suicide deaths spanning January 2001 to December 2015 from the Canadian Vital Statistics Death Database. Data from 2016 onwards were not included in the main analysis as Ontario and Yukon data were unavailable due to data-sharing agreement limitations. However, data from 2016–2019 were included in the sensitivity analyses. Seasonal variations were assessed by evaluating the timing of peak and trough monthly suicide mortality rates, and amplitude (peak-to-trough risk ratio) overall and by sex and age groups. Statistical significance was tested using the Seasonal Autoregressive Integrated Moving Average procedure.

**Results:**

With 40,405 recorded suicides, results indicated a peak in suicide rates in July (0.95 per 100,000 people), 6.74% higher than the monthly average, and a trough in February (0.78 per 100,000 people), 12.36% lower than the monthly average. The peak/trough relative risk (RR) was 1.22 (95% CI: 1.00, 1.47). Seasonality patterns were similar between sexes, but stronger and more stable among males. Seasonality patterns were similar across age groups, but the amplitude was larger among those aged 65+.

**Conclusions:**

This study confirms seasonal patterns in suicide mortality in Canada, peaking during the summer months. Such patterns are more prominent and stable among males, and its amplitude was larger among older adults. Contrary to the common perception that suicide rates peak in January, our findings show a summer peak. Future research should explore the underlying mechanisms driving these seasonal variations and their demographic differences.

## Résumé

**Objectif:**

Examiner la saisonnalité de la mortalité par suicide au Canada et explorer les différences selon le sexe et l’âge.

**Méthodes:**

Les décès mensuels par suicide survenus entre janvier 2001 et décembre 2015 ont été analysés à partir de la Base canadienne de données de l'état civil sur les décès. Les données de 2016 et des années suivantes n'ont pas été incluses dans l'analyse principale, car les données de l'Ontario et du Yukon n'étaient pas disponibles en raison de restrictions liées aux ententes de partage de données. Toutefois, les données de 2016 à 2019 ont été incluses dans les analyses de sensibilité. Les variations saisonnières ont été évaluées en déterminant le moment des taux mensuels de mortalité par suicide les plus élevés (pic) et les plus bas (creux), ainsi que l'amplitude (rapport de risques pic/creux) pour l'ensemble de la population et selon le sexe et les groupes d'âge. La signification statistique a été testée à l'aide de la procédure de moyenne mobile intégrée autorégressive saisonnière (SARIMA).

**Résultats:**

Parmi les 40 405 suicides enregistrés, les résultats indiquaient un pic des taux de suicide en juillet (0,95 pour 100 000 personnes), soit 6,74% au-dessus de la moyenne mensuelle, et un creux en février (0,78 pour 100 000 personnes), soit 12,36% en dessous de la moyenne mensuelle. Le risque relatif (RR) pic/creux était de 1,22 (IC à 95%: 1,00–1,47). Les profils de saisonnalité étaient similaires entre les sexes, mais plus marqués et plus stables chez les hommes. Les profils de saisonnalité étaient également similaires entre les groupes d’âge, mais l’amplitude était plus importante chez les personnes âgées de 65 ans et plus.

**Conclusion:**

Cette étude confirme l’existence de profils saisonniers de la mortalité par suicide au Canada, avec un pic durant les mois d’été. Ces profils sont plus prononcés et plus stables chez les hommes, et leur amplitude est plus élevée chez les personnes âgées. Contrairement à la perception courante selon laquelle les taux de suicide culminent en janvier, nos résultats montrent un pic estival. Des recherches futures devraient explorer les mécanismes sous-jacents à l’origine de ces variations saisonnières et de leurs différences démographiques.

## Introduction

Suicide is a major public health issue and a leading cause of death worldwide, with an age-standardized mortality rate of 8.9 per 100,000 people globally and 11.7 per 100,000 people in Canada (Liu et al., 2021; World Health Organization, 2025). Seasonal fluctuations in suicide mortality rates have been documented and typically feature a peak during spring or summer months and a dip during fall/winter months (Della et al., 2023; Yu et al., 2020; Trudeau, 1997; Eastwood and Peacocke, 1976). Although most studies have observed broadly consistent seasonal peaks and troughs in suicide mortality, some inconsistencies remain, which may reflect regional differences. Additionally, the aggregate seasonal pattern may obscure subgroup variations, namely by sex and age.

While sex differences in suicide mortality have been extensively documented (World Health Organization, 2025), evidence on whether sex differences are also present in seasonal patterns is inconsistent. Sex differences could be attributed to biological, environmental, and sociocultural factors (Woo et al., 2012). Males typically use more lethal methods, which are more accessible during warmer months (e.g., firearms during hunting season), potentially contributing to stronger seasonal patterns. A study in England reported a spring peak in male suicides, and spring and fall peaks in females (Meares et al., 1981). An older Ontario study using 1969–1973 data reported suicide peaks in May and October for males, and in March and October for females (Eastwood and Peacocke, 1976). More recent evidence suggests sex differences in seasonal variation, with larger amplitudes (i.e., a larger difference between peak and trough suicide rates) among females than males in the United Kingdom (UK) and the United States (US), but not in Canada (Yu et al., 2020). However, Canadian findings were based on urban cities, excluding rural and northern regions. Altogether, findings on sex differences in Canadian seasonal suicide patterns are over three decades old and limited to urban cities.

Seasonal patterns may also differ across age groups (World Health Organization, 2025). Examining the seasonality of suicide by age group is important from a life course perspective, as the timing and nature of stressors vary across different stages of life. For example, youth may be more affected by stressors tied to the school year (Hansen & Lang, 2011; Matsubayashi et al., 2016), such as academic pressures or transitions in peer relationships, whereas older adults may be more influenced by seasonal factors such as social isolation or changes in daily routines in colder seasons, or greater health challenges due to higher temperatures in the summer (Matsubayashi et al., 2016; Woo et al., 2012). Evidence on age-related seasonal differences in suicide mortality remains mixed. A review found that studies reported peaks in spring, fall or winter months among younger individuals (Woo et al., 2012), while more recent research identified peaks in fall months only (To et al., 2024) or dual peaks in spring and fall months (Matsubayashi et al., 2016). Among older adults, some studies observed peaks in summer months (To et al., 2024; Woo et al., 2012), whereas a study from British Columbia reported a September peak (Marion et al., 1999). Regarding troughs, suicide mortality among younger individuals was lowest in June (To et al., 2024) or during summer months (Hansen & Lang, 2011; Matsubayashi et al., 2016). For older adults, rates were the lowest in winter months (Marion et al., 1999; To et al., 2024). Cross-national analyses using 1986–1999 data further found larger seasonal variability among older than younger individuals (Yu et al., 2020). While evidence supports age-related seasonal differences in suicide mortality rates, Canadian findings remain outdated or geographically limited, and the absence of a life course perspective could obscure important differences across life stages (Eastwood and Peacocke, 1976; Marion et al., 1999; Komar et al., 2022; Malla & Hoenig, 1979; Trudeau, 1997; Tugaleva et al., 2016; Yu et al., 2020).

Canada’s unique geographic and climatic conditions, including extreme seasonal variations in daylight and temperature, may uniquely influence suicide mortality rate patterns compared to other regions. Canadian research remains outdated, using pre-2010 data, and is often limited to specific regions or urban population, limiting generalizability to the Canadian context. This is particularly problematic for examining sex and age differences, where available evidence relies on data over three decades old. Collectively, these gaps underscore the need for updated, nationally representative research. Accordingly, the present study has two objectives: (1) to analyze seasonal suicide mortality rates in Canada using nationally representative data, and (2) to investigate potential sex- and age-specific seasonal patterns in suicide mortality rates.

## Methods

### Data source

Suicide deaths from January 2001 to December 2019 for individuals aged 15 and older were extracted from the Canadian Vital Statistics Death Database (CVSD) (Statistics Canada, 2024). Individuals under 15 were excluded due to model convergence issues caused by insufficient time observations. The CVSD collects demographic and medical data on all deaths across Canadian provinces and territories annually and monthly (Statistics Canada, 2024). Data from 2001–2015 were available from all provinces and territories, except Québec due to a lack of data-sharing agreement between the Public Health Agency of Canada (PHAC) and Québec to access mortality data. Data from 2016 onwards were not included in the main analysis as Ontario (representing nearly 40% of the Canadian population) and Yukon data were unavailable due to data-sharing agreement limitations. However, data from 2016–2019 were included in the sensitivity analyses. Annual population estimates from Statistics Canada were used for both overall and age- and sex-stratified analyses (Statistics Canada, 2024a; Statistics Canada, 2024b).

Suicide deaths were identified using the 10th revision of the International Classification of Diseases (ICD-10) codes for intentional self-harm (X60-X84, Y87.0). Individuals coded as “other” or “unknown” for sex were excluded. Using the life course approach, age was categorized into 15–24 (emerging adulthood), 25–44 (young adulthood), 45–64 (mid-adulthood), and 65 + (older adulthood) (Elder et al., 2003).

### Statistical analysis

Analyses were conducted in SAS Enterprise Guide 7.1 for the overall population and then stratified by sex and age groups. Suicide mortality rates per 100,000 people were calculated per month from 2001–2015 using annual population estimates as the denominator. The mean monthly suicide rate was then estimated, and the shape of the monthly suicide mortality pattern, including peaks (maximum) and troughs (minimum), was described. Percent differences in monthly suicide mortality rates compared to the annual average were calculated. To characterize the amplitude of seasonal variation in suicide mortality, the peak/trough relative risk (RR) was calculated by dividing the suicide mortality rate during the peak month by the trough month. This method has been used to compare suicide seasonal variations across countries, including the US and Sweden (Reutfors et al., 2009; Yu et al., 2020). Age was not standardized, as our analysis did not involve comparisons across different populations (e.g., across provinces).

We applied a seasonal autoregressive integrated moving average (SARIMA) model to examine the seasonal patterns of suicide rates and their stability over time. SARIMA models are used in applied time-series analyses to characterize seasonality effects (e.g., patterns of suicide) (SAS Institute Inc., Cary, NC, USA) (Reutfors et al., 2009). We used the X13 procedure in SAS, which applies an automatic model selection procedure to the monthly suicide mortality rates.

Tests were conducted to assess seasonality: (a) the Stable Seasonality Test, (b) the Moving Seasonality Test, and (c) the Combined Seasonality Test. The Stable Seasonality Test evaluates the consistency of seasonal patterns across years using an F-test to compare between-month variation to within-month variation (Ladiray & Queeneville, 2012). A significant result (*p*_*S*_ <0.001) indicates stable seasonality. The Moving Seasonality Test then determines if seasonal patterns evolve over time through an F-test that assesses the interaction between month and year. A significant F-test (*p*_*M*_ <.05) indicates that the seasonal pattern is changing year to year. The Combined Seasonality Test integrates results from the Stable Seasonality Test and the Moving Seasonality Test using two statistics, T1 and T2, which are derived from F-statistics of the respective tests (see Table 3 footnotes for the formulas). When both T1 and T2 < 1, seasonality is present. If either T1 ≥ 1 or T2 ≥ 1, seasonality is probably not present and if both T1 and T2 are ≥ 1, seasonality is not present. When both conditions for identifiable seasonality are met (T1 < 1 and T2 < 1), a Kruskal-Wallis chi-squared test is conducted at the 1% significance level for final confirmation of identifiable seasonality (Lothian & Morry, 1978).

### Additional analysis

We conducted a sensitivity analysis using 2016–2019 data, excluding Ontario, the Yukon and Québec, to determine whether the observed seasonal patterns remained consistent during this time period. The denominator in this analysis excluded Ontario, the Yukon, and Québec data.

## Results

### Seasonal variation in suicide mortality overall

**Fig. 1:**
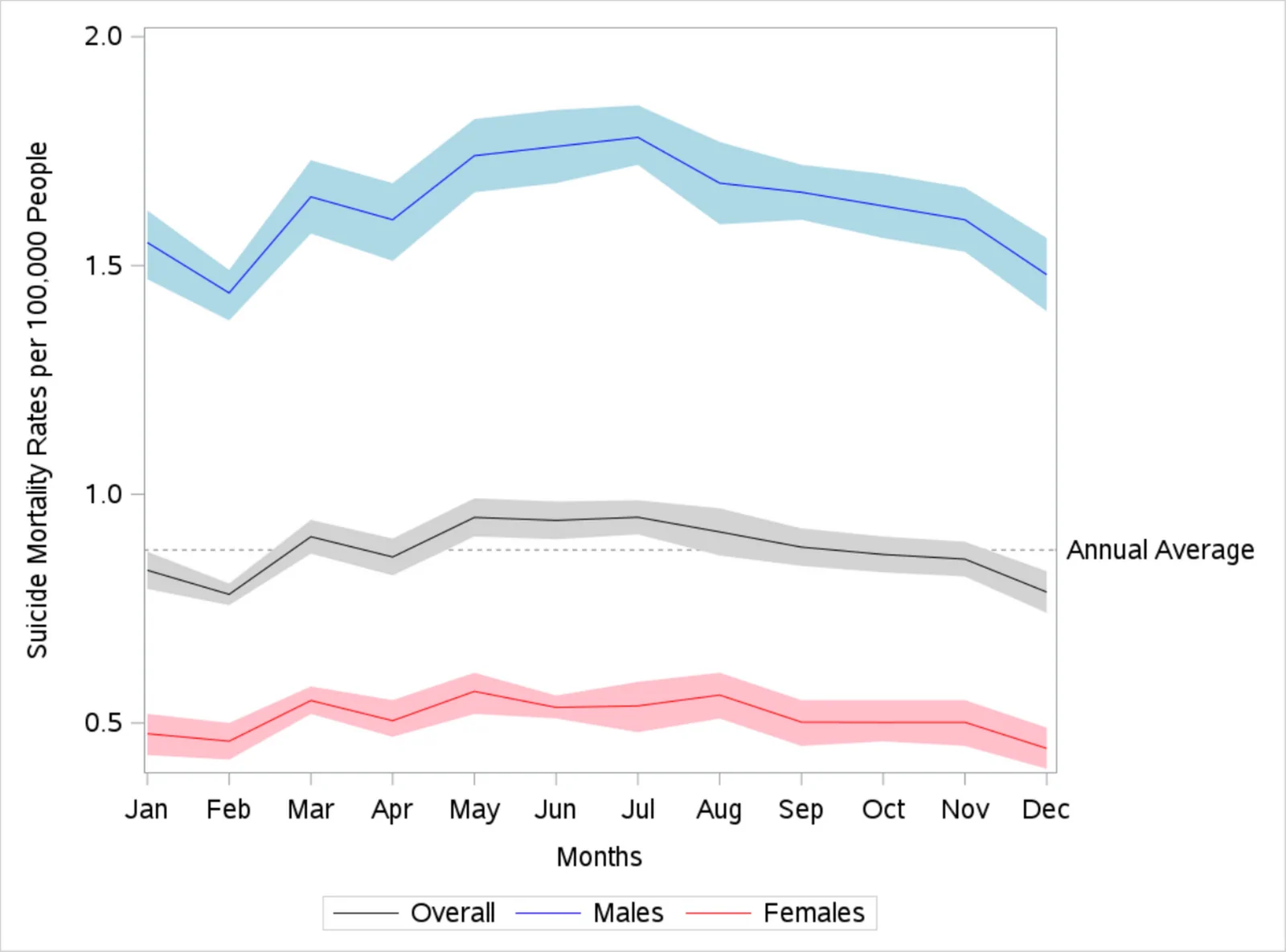
Average monthly suicide mortality rates per 100,000 people in Canada, excluding Québec, by sex, 2001–2015

The seasonal patterns in monthly suicide mortality rates are presented (Fig. 1, Tables 1 and 2). Among 40,405 deaths from suicide, the average monthly suicide mortality rates peaked in July (0.95 per 100,000 people), 6.74% above the monthly average (0.89 per 100,000 people), with elevated rates in March, May, June, and July. The mortality rate was the lowest in February (0.78 per 100,000 people), 12.36% below the monthly average. The peak/trough RR was 1.22 (95% CI, 1.00, 1.47) (Table 2), suggesting the suicide mortality rate was 22% greater in July compared to February. In the SARIMA model, the combined seasonality test confirmed the presence of suicide rate seasonality patterns (T1 < 1 and T2 < 1, *p* <.001). The Stable Seasonality Test showed that the intrayear variation repeated consistently (*p* <0.001). The Moving Seasonality Test showed that seasonality did not significantly vary year to year (*p* =0.54).

**Table 1:** Monthly average of suicide mortality rates by month per 100,000 people in Canada, by sex and age, excluding Québec, 2001–2015

	January	February	March	April	May	June	July	August	September	October	November	December	Monthly average
Overall Rate per 100,000 (95% CI)	0.83 (0.79, 0.88)	0.78 (0.76, 0.81)	0.91 (0.87, 0.95)	0.86 (0.82, 0.91)	0.95 (0.90, 0.99)	0.94 (0.90, 0.99)	0.95 (0.91, 0.99)	0.92 (0.86, 0.97)	0.88 (0.84, 0.93)	0.87 (0.83, 0.91)	0.86 (0.82, 0.90)	0.79 (0.74, 0.84)	0.89 (0.85, 0.90)
Sex													
Males	1.55 (1.47, 1.62)	1.44 (1.38, 1.49)	1.65 (1.57, 1.73)	1.60 (1.51, 1.68)	1.74 (1.66, 1.82)	1.76 (1.68, 1.84)	1.78 (1.72, 1.85)	1.68 (1.59, 1.77)	1.66 (1.60, 1.72)	1.63 (1.56, 1.70)	1.60 (1.53, 1.67)	1.48 (1.40, 1.56)	1.63 (1.61, 1.66)
Females	0.48 (0.43, 0.52)	0.46 (0.42, 0.50)	0.55 (0.52, 0.58)	0.51 (0.47, 0.55)	0.57 (0.52, 0.61)	0.53 (0.51, 0.56)	0.54 (0.48, 0.59)	0.56 (0.51, 0.61)	0.50 (0.45, 0.55)	0.50 (0.46, 0.55)	0.50 (0.45, 0.55)	0.44 (0.40, 0.49)	0.51 (0.49, 0.54)
Age group													
15–24	0.84 (0.76, 0.93)	0.82 (0.76, 0.87)	0.84 (0.78, 0.90)	0.85 (0.77, 0.94)	0.97 (0.89, 1.05)	1.00 (0.88, 1.11)	0.88 (0.80, 0.97)	0.96 (0.85, 1.07)	0.95 (0.85, 1.05)	0.89 (0.79, 0.98)	0.83 (0.77, 0.88)	0.78 (0.70, 0.86)	0.88 (0.86, 0.91)
25–44	1.02 (0.95, 1.09)	0.94 (0.88, 0.99)	1.06 (0.98, 1.14)	1.01 (0.94, 1.09)	1.12 (1.05, 1.19)	1.13 (1.06, 1.20)	1.20 (1.13, 1.27)	1.09 (1.01, 1.17)	1.02 (0.94, 1.09)	1.04 (0.98, 1.10)	1.02 (0.97, 1.08)	0.96 (0.91, 1.01)	1.05 (1.03, 1.07)
45–64	1.18 (1.10, 1.25)	1.11 (1.03, 1.18)	1.31 (1.25, 1.37)	1.22 (1.15, 1.29)	1.31 (1.23, 1.38)	1.27 (1.19, 1.36)	1.30 (1.23, 1.37)	1.26 (1.17, 1.35)	1.23 (1.14, 1.33)	1.23 (1.15, 1.30)	1.23 (1.13, 1.33)	1.12 (1.04, 1.21)	1.23 (1.21, 1.25)
65 +	0.74 (0.66, 0.81)	0.69 (0.62, 0.77)	0.90 (0.85, 0.95)	0.86 (0.77, 0.95)	0.98 (0.91, 1.05)	0.96 (0.89, 1.02)	0.96 (0.87, 1.05)	0.97 (0.88, 1.06)	0.91 (0.82, 1.01)	0.83 (0.74, 0.92)	0.88 (0.80, 0.95)	0.74 (0.64, 0.84)	0.87 (0.84, 0.89)

Source: Canadian vital statistics death database

Abbreviations: CI, confidence intervals

**Table 2:** Seasonal patterns of monthly suicide mortality rates per 100,000 people by sex and age in Canada, excluding Québec, 2001–2015

Category	Peak month	Trough month	Peak rate (95%CI) (per 100,000 people)	Trough rate (95%CI) (per 100,000 people)	Peak/trough relative risk (95%CI)
Overall population	July	Feb	0.95 (0.91. 0.99)	0.78 (0.76, 0.80)	1.22 (1.00, 1.47)
Sex					
Males	July	Feb	1.78 (1.72, 1.85)	1.44 (1.38, 1.49)	1.24 (1.01, 1.52)
Females	May	Dec	0.57 (0.52, 0.58)	0.44 (0.40, 0.49)	1.28 (0.80, 2.05)
Age group					
15–24	June	Dec	1.00 (0.88, 1.11)	0.78 (0.70, 0.86)	1.28 (0.71, 2.31)
25–44	July	Feb	1.20 (1.13, 1.27)	0.94 (0.88, 0.99)	1.28 (0.92, 1.79)
45–64	Mar	Feb	1.31 (1.25, 1.37)	1.11 (1.03, 1.18)	1.18 (0.85, 1.64)
65 +	May	Feb	0.98 (0.91, 1.05)	0.69 (0.62, 0.77)	1.41 (0.85, 2.33)

Source: Canadian vital statistics death database

Abbreviations: CI, confidence intervals

### Seasonal variation in suicide mortality by sex

The average monthly suicide mortality rate was 1.63 per 100,000 people among males and 0.51 per 100,000 people among females (Fig. 1; Table 1). Among males, suicide mortality rates peaked in July (1.78 per 100,000 people), 9.20% above the male monthly average, with elevated rates from May to August. The lowest rate was in February (1.44 per 100,000 people), 11.66% below the male monthly average. In females, the suicide mortality rates peaked in May (0.57 per 100,000 people), 11.76% above the female monthly average, with elevated rates from May to August. The trough was in December (0.44 per 100,000 people), 13.73% below the female monthly average. The peak/trough RR was 1.24 (95% CI, 1.01, 1.52) among males and 1.28 (95% CI, 0.80, 2.05) among females (Table 2), suggesting similar seasonality amplitude patterns.

The Stable Seasonality Test demonstrated that seasonality patterns were consistent across years (Table 3), with males showing a stronger and more stable effect (F_S_ = 12.46, *p*_*S*_ <0.001) than females (F_S_ = 4.35, *p*_*S*_ <0.001). The Moving Seasonality Test found no moving seasonality in males (F_M_ = 0.65, *p*_*M*_ =0.82), but significant variations in females (F_M_ = 2.25, *pM* =0.008), indicating the seasonal pattern in females was fluctuating from year to year. The Combined Seasonality Test found that the patterns were identifiable in males (T1 < 1, T2 < 1, *p*_*KW*_ <0.001) but not in females (T1 ≥ 1, T2 ≥ 1).

**Table 3:** Tests of seasonality using X13 ARIMA model combined seasonality test for the overall population in Canada, excluding Québec, by sex and by age groups, 2001–2015

Category	Stable seasonality test	Moving seasonality test	Combined seasonality test			
			T1^a^	T2^b^	KW test	Identifiable seasonality
Overall population	Fs = 16.14, *p*_*S*_ < 0.001	F_M_ = 0.92, *p*_*M*_ = 0.54	0.43	0.17	χ^2^ = 95.37, *p*_*kw*_ < 0.001	Present (T1 and T2 < 1)
By sex						
Males	Fs = 12.46, *p*_*S*_ < 0.001	F_M_ = 0.65, *p*_*M*_ = 0.82	0.56	0.16	χ^2^ = 85.37, *p*_*kw*_ < 0.001	Present (T1 and T2 < 1)
Females	Fs = 4.35, *p*_*S*_ < 0.001	F_M_ = 2.25, *p*_*M*_ = 0.008	1.61	1.55	NA	Not present (T1 and T2 ≥ 1)
By age group						
15–24	Fs = 3.00, *p*_*S* =_0.001	NA	NA	NA	NA	Not present (ps = 0.001)
25–44	Fs = 6.48, *p*_*S*_ < 0.001	F_M_ = 0.74, *p*_*M*_ = 0.74	1.08	0.34	NA	Probably not Present (T1 or T2 ≥ 1)
45–64	Fs = 4.01, *p*_*S*_ < 0.001	F_M_ = 1.65, *p*_*M*_ = 0.07	1.75	1.24	NA	Not present (T1 and T2 ≥ 1)
65+	Fs = 3.87, *p*_*S*_ < 0.001	F_M_ = 0.58, *p*_*M*_ = 0.88	1.07	0.27	NA	Probably not present (T1 or T2 ≥ 1)

Source: Canadian vital statistics death database

Abbreviations: F_S_, F-test statistic for the Stable Seasonality Test; F_M_, F-test statistic for the Moving Seasonality Test; *p*_*S*_, p-value for the Stable Seasonality Test; *p*_*M*_, p-value for the Moving Seasonality Test; *p*_*KW*_, p-value for the Kruskal–Wallis test; T1 and T2, statistics derived from the Stable Seasonality and Moving Seasonality Test F-test statistics; χ^2^, Chi-square statistic; KW, Kruskal–Wallis test

^a^The formula to calculate T1 statistic is T1 = 7/F_S_

^b^The formula to calculate T2 statistic is T2 = 3*F_M_/F_S_

### Seasonal variation in suicide mortality by age groups

The age-stratified SARIMA model excluded individuals under 15 years, due to model convergence issues because of insufficient time observations. The average monthly suicide mortality rates were highest among ages 45–64 (1.23 per 100,000 people), followed by ages 25–44 (1.05 per 100,000), 15–24 (0.88 per 100,000), and 65 + (0.87 per 100,000) (Table 1). Seasonal variation was evident across all age groups, with rates peaking in the summer months and reaching their lowest level in winter months (i.e., February or December). An exception was observed among adults aged 45–64, where the peak of suicide mortality rates occurred in March rather than in the summer months.

Specifically, those aged 15–24, 25–44, and 65 + showed a late spring/summer peak in suicide mortality rates. Mortality rates peaked in June for ages 15–24 (1.00 per 100,000 people), 13.64% above the stratum-specific monthly average. Ages 25–44 reported a July peak (1.20 per 100,000 people), 14.29% above the stratum-specific monthly average. Ages 65 + reported a May peak (0.98 per 100,000), 12.64% above the stratum-specific monthly average. Ages 45–64 reported a March peak (1.31 per 100,000 people), 6.50% above the stratum-specific monthly average. The mortality rates were lowest in December for ages 15–24 (0.78 per 100,000 people), 11.36% below the stratum-specific monthly average. Those aged 25–44, 45–64 and 65 + all reported the lowest suicide mortality rates in February (0.94, 1.11, and 0.69 per 100,000 people, respectively), 10.48%, 9.96%, and 20.69% below the stratum-specific monthly average, respectively. The peak/trough RR varied across age groups and was highest among those aged 65 + (Table 2), indicating greater seasonal variation among older adults, though not statistically significant.

The Stable Seasonality Test showed that all age groups showed consistent seasonal patterns across years (*p*_*S*_ < 0.001), except for ages 15–24 (*p*_*S*_ =0.001). The Moving Seasonality Tests were not significant for ages 25–44, 45–64, and 65 +, indicating seasonal patterns did not vary year to year. The Combined Seasonality Test of the SARIMA model suggested that seasonal patterns were probably not present for those aged 25–44 and 65 + (T1 > 1 or T2 > 1) and were not present at all for ages 45–65 (T1 > 1 and T2 > 1) (Table 3).

### Sensitivity analysis

Seasonal patterns using 2016–2019 data showed that suicide mortality rates were similar to our main analysis using 2001–2015 data, with July peaks and February troughs. However, suicide mortality rates were higher in 2016–2019 (July peak: 1.20; February trough: 1.05 per 100,000) than in 2001–2015 (peak: 0.95, trough: 0.75 per 100,000) (results available upon request).

## Discussion

This study examined seasonal suicide mortality rates in Canada and potential sex- and age-specific seasonal patterns. From 2001–2015, suicide mortality rates exhibited seasonal patterns in Canada, with late spring (May) and early summer months peaks (July), and troughs in winter (February). This pattern was comparable between sexes, but stronger and more stable among males. Suicide rates peaked in the summer months across age groups, except ages 45–64, which peaked in March. Older adults (65 +) exhibited the greatest amplitude of seasonal variation, though not statistically significant.

### Overall seasonal patterns

Seasonal suicide mortality patterns, with a summer peak and winter trough, have been observed in various countries and in earlier Canadian studies. Prior research from the UK, US, and Canada has generally shown a spring peak (April, May) and a winter trough (December–February) in suicide mortality rates (Cavanagh et al., 2016; Eastwood & Peacocke, 1976; Yu et al., 2020). Meanwhile, findings from Newfoundland and Labrador reported a summer peak (July) and a winter trough (November) (Komar et al., 2022).

Our study showed summer peaks (July) in suicide mortality rates, later than the spring peaks (April, May) reported in earlier Canadian, UK and US studies (Cavanagh et al., 2016; Eastwood & Peacocke, 1976; Yu et al., 2020). Troughs in winter months align with prior Canadian, UK, and US studies (Cavanagh et al., 2016; Eastwood & Peacocke, 1976; Komar et al., 2022; Yu et al., 2020). However, the months of peaks differed slightly from previous studies, likely due to context: (1) our use of national data (including most provinces and territories) versus urban samples in Canada (Yu et al., 2020), and (2) previous studies relied on data specific to the US, UK or province (Cavanagh et al., 2016; Eastwood & Peacocke, 1976; Yu et al., 2020).

We found a peak-to-trough RR of 1.22 in Canada, indicating suicide mortality rates were 22% higher in the peak versus the trough month, aligning with earlier research where comparable RRs have been reported in Canada (1.23) and the US (1.15) (Yu et al., 2020). We also found that seasonal highs were 6.74% above and troughs were 12.36% below the monthly average. Similarly, a 1997 Canadian study reported seasonal highs 9% above and troughs 14% below the annual average (Trudeau, 1997). Altogether, our findings extend prior Canadian work by providing national evidence (all provinces and territories except Québec), showing that the overall seasonal pattern of a summer peak and winter trough persists.

### Results by sex

Suicide mortality rates peaked in summer (July) for males and spring (May) for females, aligning with prior studies using 1960–1984 Canadian data showing peaks in summer (June) for males and spring (May) for females (Yu et al., 2020).

In our study, RRs were comparable across sexes, but seasonal patterns were stronger among males than females. This aligns with earlier US and UK studies that also found similar RRs between sexes (Chew & McCleary, 1995), and Australian data showed stronger seasonal patterns in males (Burns, 2016). Other factors, such as biological, environmental, and sociocultural influences, may contribute to the higher suicide rates among males compared with females (Woo et al., 2012). A contributing factor to the stronger seasonal patterns observed among males may be their more frequent use of lethal methods of suicide (e.g., firearms), which also tend to be more accessible during warmer months (e.g., during hunting season). One interesting finding was that moving seasonality was significant among females but not males, indicating seasonal patterns in females are less consistent year to year. An Italian study also found more significant changes in seasonal trends among females (Preti & Lentini, 2016). Altogether, our Canadian study revealed sex-specific seasonal variations in suicide mortality rates.

### Results by age group

**Fig. 2:**
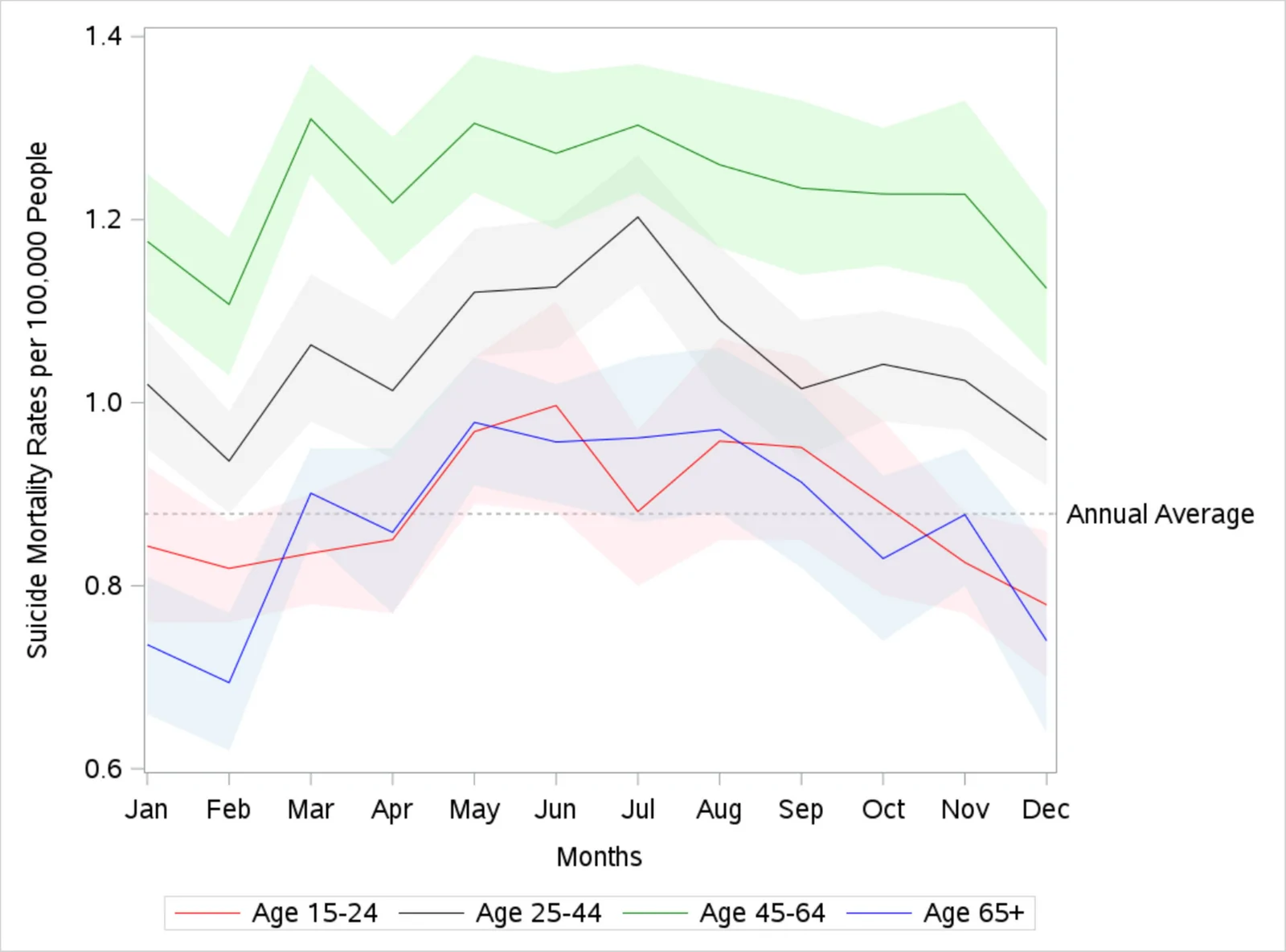
Average monthly suicide mortality rates per 100,000 people in Canada, excluding Québec, by age group, 2001–2015

Suicide mortality rates peaked in June for ages 15–24, July for 25–44, March for 45–64, and May for 65+. A study using 1981–1991 British Columbia (BC) data reported June peaks for ages 10–64, but September peaks for ages 65 + (Marion et al., 1999). This difference may reflect BC’s distinct climate compared to other provinces. Nonetheless, suicide mortality rates were lowest in winter (February or December) across all age groups, consistent with the BC study (Marion et al., 1999). Interestingly, no spike in suicide mortality rates was observed among ages 15–24 (Fig. 2), possibly reflecting adaptation from school breaks coinciding with day light saving time (DST) onset.

Consistent with prior literature (Marion et al., 1999; Yu et al., 2020), we also found the amplitude of seasonality patterns was highest in ages 65 +, followed by 15–24, 25–44, and then 45–64. Biological and environmental factors may underlie this association: older adults’ reduced ability in body temperature regulation increases vulnerability to seasonal temperature fluctuations, impairing health and mobility, and elevating suicide risk (Burns, 2016). Using a life course lens, our age-stratified analysis showed seasonal patterns that vary across age groups, reflecting biological and environmental differences.

### Strengths and limitations

One strength of this study is its use of data covering most of Canada’s population (excluding Québec), making these results broadly representative. We also analyzed sex- and age-specific seasonality patterns, and temporal patterns in seasonality using 15 years of data.

This study also has several limitations. First, the analysis did not include Quebec throughout and Ontario and Yukon after 2015 due to a lack of data-sharing agreement with PHAC, potentially limiting generalizability. This was not related to the legalization of medical assistance in dying (MAiD). In particular, MAiD deaths are coded separately from suicide in the Canadian Vital Statistics Death (CVSD) database and therefore did not affect our analyses. However, the introduction of MAiD may reflect a broader contextual shift in end-of-life decision-making that may be relevant for future research. Second, the power to detect seasonality patterns was limited for age-stratified analyses due to the small number of suicide deaths in subgroups. Insufficient time observations for individuals aged < 15 led to model convergence issues. Third, we did not account for external factors (e.g., inflation, economic recession, unemployment) with seasonal patterns that could amplify or attenuate observed suicide patterns. Fourth, we did not compare provinces and territories. Variations in DST practices and solar time alignment (e.g., Alberta’s late sunsets in winter due to its position west of solar time, and Saskatchewan’s non-observance of DST) could contribute to provincial variation in the timing or intensity of seasonal suicide peaks (De Koninck et al., 2024). Finally, analyses of other sociodemographic factors related to suicide (e.g., rurality, occupation) were not possible due to data availability constraints.

### Future directions

Future studies should examine seasonal suicide patterns by suicide method, regions, and rurality (e.g., rural vs. urban) to determine subgroup differences. Future research is needed to explore mechanisms underlying sex- and age-specific seasonal suicide patterns by integrating other data sources (e.g., weather patterns, economic indicators). Future research should examine how provincial differences in DST practices and solar time alignment affect seasonal suicide mortality and explore the seasonality patterns of suicide-related behaviors by utilizing other datasets (e.g., hospitalization, emergency department visits, suicide crisis line utilization). Given that males are more likely to die by suicide and females are more often hospitalized (Meares et al., 1981), different suicide-related behaviors may have unique seasonal patterns by subgroups.

## Conclusions

This nationwide analysis of suicide mortality in Canada (excluding Québec) from 2001 To 2015 shows clear seasonal patterns, with summer peaks and winter troughs. These patterns occur across sexes and age groups but are more pronounced and stable among males and more variable in older adults aged 65 +. Future suicide research should examine mechanisms driving seasonal patterns and interactions between demographic and environmental factors on the seasonality of suicide.

## Data Availability

Not applicable.

## Code availability

Code would not be available.
